# Photocatalytic degradation of ciprofloxacin using CuFe_2_O_4_@methyl cellulose based magnetic nanobiocomposite

**DOI:** 10.1016/j.mex.2019.12.005

**Published:** 2019-12-09

**Authors:** Fatemeh Tamaddon, Alireza Nasiri, Ghazal Yazdanpanah

**Affiliations:** a Department of Chemistry, Faculty of Science, Yazd University, Yazd 89195-741, Iran; b Environmental Health Engineering Research Center, Kerman University of Medical Sciences, Kerman, Iran

**Keywords:** Photocatalytic method to ciprofloxacin degradation with CuFe_2_O_4_@MC synthesised as a novel nanobiomagnetic photocatalyst, Ciprofloxacin, Methyl cellulose, Copper ferrite, Photocatalytic degradation, Nanobiomagnetic photocatalyst

## Abstract

Herein, magnetically separable CuFe_2_O_4_@methyl cellulose (MC) as a novel magnetic nanobiocomposite photocatalyst was synthesized with a facile, rapid, green, and new microwave-assisted method. After that, CuFe_2_O_4_@MC was characterized with FESEM, EDS, FT-IR, XRD, TGA, and VSM techniques. To measure CuFe_2_O_4_@MC photocatalytic activity, ciprofloxacin (CIP) removal ability of CuFe_2_O_4_@MC was investigated under the conditions such as initial CIP concentrations (3, 5, 7, and 9 mg/L), pHs (3, 7, and 11), photocatalyst loadings (0.025, 0.05, 0.1, 0.2, 0.3, and 0.4 g), and irradiation time (15, 30, 45, 60, 75, and 90 min). Kinetic process was evaluated with the *pseudo-*first order and the *Langmuir*-*Hinshelwood* models. CIP concentration was measured with high performance liquid chromatography (HPLC). The maximum CIP removal efficiency in the optimal conditions which contained pH = 7, CIP initial concentration of 3 mg/L, photocatalyst loading of 0.2 g, and at irradiation time 90 min was achieved 72.87 % and 80.74 % from real and synthetic samples, respectively. Also, COD removal efficiency in the optimal conditions was achieved 68.26 %. Furthermore, the CuFe_2_O_4_@MC reusability and chemical stability were examined and 73.78 % of CIP was degraded after the fourth cycle.

**Advantages of this technique were as follows:**

• CuFe_2_O_4_@MC as a new nanobiomagnetic photocatalyst was synthesized with a facile, fast, and green method and were characterized with FESEM, EDS, FT-IR, XRD, TGA, and VSM techniques.
• Ferromagnetic property and pure-phase spinel ferrites of CuFe_2_O_4_@MC were confirmed and significant photocatalytic activity of CuFe_2_O_4_@MC was observed.
• Easily gathering, reusability and good chemical stability were interests of this nanobiomagnetic photocatalyst.

## Graphical abstract

Photodegradation of ciprofoxacin by CuFe_2_O_4_@MC

**Figure:**
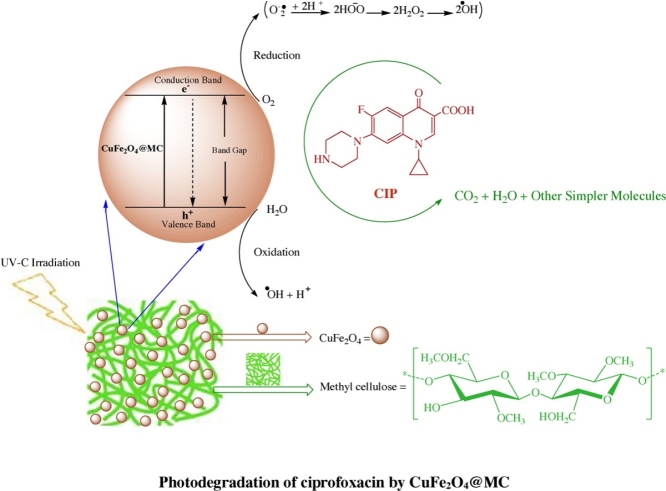

**Specification Table**

**Table:** 

Subject Area:	Environmental Sciences
More specific subject area:	Chemical engineering in environmental sciences
Method name:	Photocatalytic method to ciprofloxacin degradation with CuFe_2_O_4_@MC synthesised as a novel nanobiomagnetic photocatalyst
Name and reference of original method	Nasiri A, Tamaddon F, Mosslemin M H, Amiri Gharaghani M, Asadipour A. Magnetic nanobiocomposite CuFe_2_O_4_@methylcellulose prepared as a new nano-photocatalyst for degradation of ciprofloxacin from an aqueous solution. Environmental Health Engineering and Management Journal. (2019); 6(1):41-51. DOI: 10.15171/EHEM.2019.05
Resource availability	NA

## Method details

Pharmaceutical pollutants could enter to the aquatic environment from residual part of pharmaceutical industry, hospital wastewater, and human waste disposal. Antibiotics are one of these pollutants, which metabolize imperfectly and enter to various sources of water [1]. Ciprofloxacin (CIP) is the most applied antimicrobial and anti-inflammatory antibiotics that uses in infection diseases treatment. Due to the high-dose usage of CIP and other antibiotics, serious environmental damages were occurred by accumulation of these antibiotics in wastewater, plants, and animal cells [2,3]. Because of the low biodegradability, the conventional volatilization, adsorption, sedimentation, coagulation, and biological methods are not effective ways for removal of CIP and other antibiotics from the aquatic environment [[4], [5], [6], [7]]. Thus, new efficient methods for CIP removal are desirable. Recently, the advanced oxidation processes (AOPs) with heterogeneous photocatalysts has been regarded as a promising option for CIP-containing wastewaters treatment [8]. The AOPs with photocatalysts involves generation of the highly reactive hydroxyl radical to eliminate of the organic pollutants with oxidative degradation. In the heterogeneous AOPs, the UV-irradiation as energy source excites photoactive semiconductors [9] which causes electron transfer from the valence band to the conductive band, electron-hole (h^+^) pairs production, and hydroxyl radical (OH^•^) or other oxidizing radicals were generated for oxidation of hazardous materials [10]. Moreover, hydroxyl radical (OH^•^) production, photocatalysis efficiency, response to light, and photocatalytic activity of photocatalysts increase using nanophotocatalysts, due to the smaller size, greater surface area, the higher diffusion power, and superior behavior of nanomaterials [11].

Photocatalytic degradation by using metal oxides and metal spinel ferrites such as CoFe_2_O_4_, ZnFe_2_O_4_, and CuFe_2_O_4_ as photocatalyst is one of the effective methods for antibiotic removal from aqueous solutions [[12], [13], [14], [15], [16], [17], [18], [19], [20], [21]]. The photocatalytic removal process with nanophotocatalysts, which has been reported in some studies, was pH-sensitive. Also for increase degradation efficiency a chemical oxidant such as H_2_O_2_ has been used [22,23]. To prevent damage to the environment, using green processes for photocatalysts synthesis is preferred. Recently, bio-polymers as cellulose, carboxy methyl cellulose, chitosan, natural gums, agar, gelatin, chitin, collagen etc. for many reasons such as abundance in nature and multi-functionality have attracted attention [24]. These polysaccharides due to numerous properties such as non-toxicity, biocompatibility, flexibility, high functionality and ease of processing have many applications in the water treatment [25,26]. Due to, previous studies literature review, there is no research about the CIP removal using CuFe_2_O_4_@methylcellulose as a magnetic nanobiocomposite photocatalyst from aqueous solutions. CuFe_2_O_4_@methylcellulose is a new class of biocomposites, which built from organic linkers and inorganic metal nodes through coordination bonds [21]. Therefore, a magnetically separable CuFe_2_O_4_@methyl cellulose (MC) photocatalyst was designed with a facile, fast, and green microwave-assisted method.

**Fig. 1:**
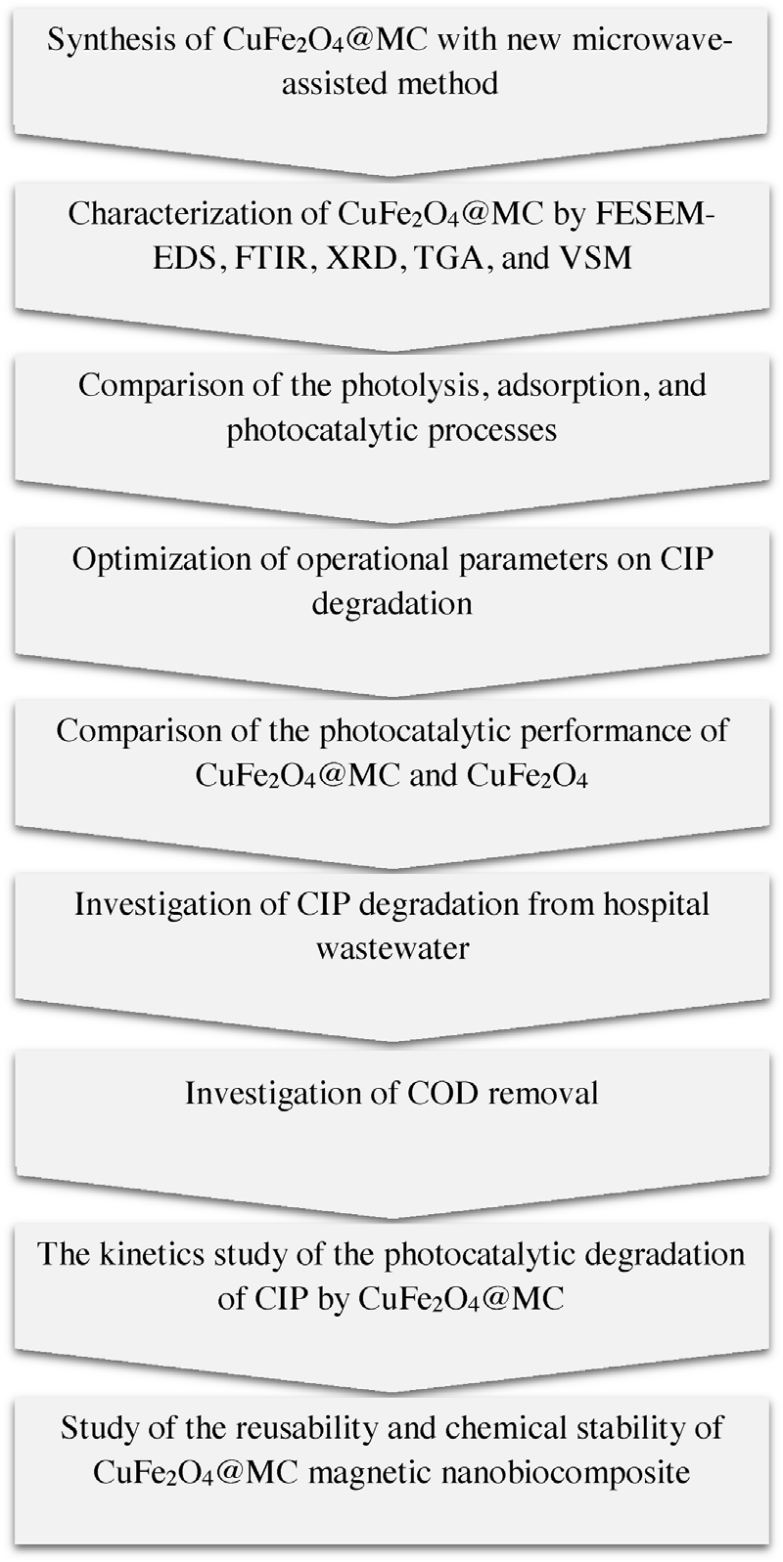
Research steps.

The research steps were as follows: at first, CuFe_2_O_4_@MC was prepared with a new microwave-assisted method and characterized. After that, the photolysis, adsorption, and photocatalytic processes were compared with each other. Then the operational parameters effects on the CIP removal efficiency were evaluated and optimized. In the next step, comparison of the photocatalytic performance of CuFe_2_O_4_@MC and CuFe_2_O_4_ was done. Also, in the optimal conditions, the CIP degradation from hospital wastewater and the COD removal were investigated. Finally, the CIP photocatalytic removal kinetics were evaluated and CuFe_2_O_4_@MC reusability and chemical stability were obtained. The study stages have been shown in the Fig. 1.

## Chemicals

FeCl_3_.6H_2_O, CuCl_2_.2H_2_O, NaOH, and methyl cellulose (MC) were purchased from Merck Company (Germany) and used without more purification. Also, CIP (99 % purification) was purchased from Tamad Pharmaceutical Company (Tehran, Iran). All the materials were in analytical grade and used without further purification. Deionized water was used during the tests.

## Synthesis and characterization of CuFe_2_O_4_@MC

**Fig. 2:**
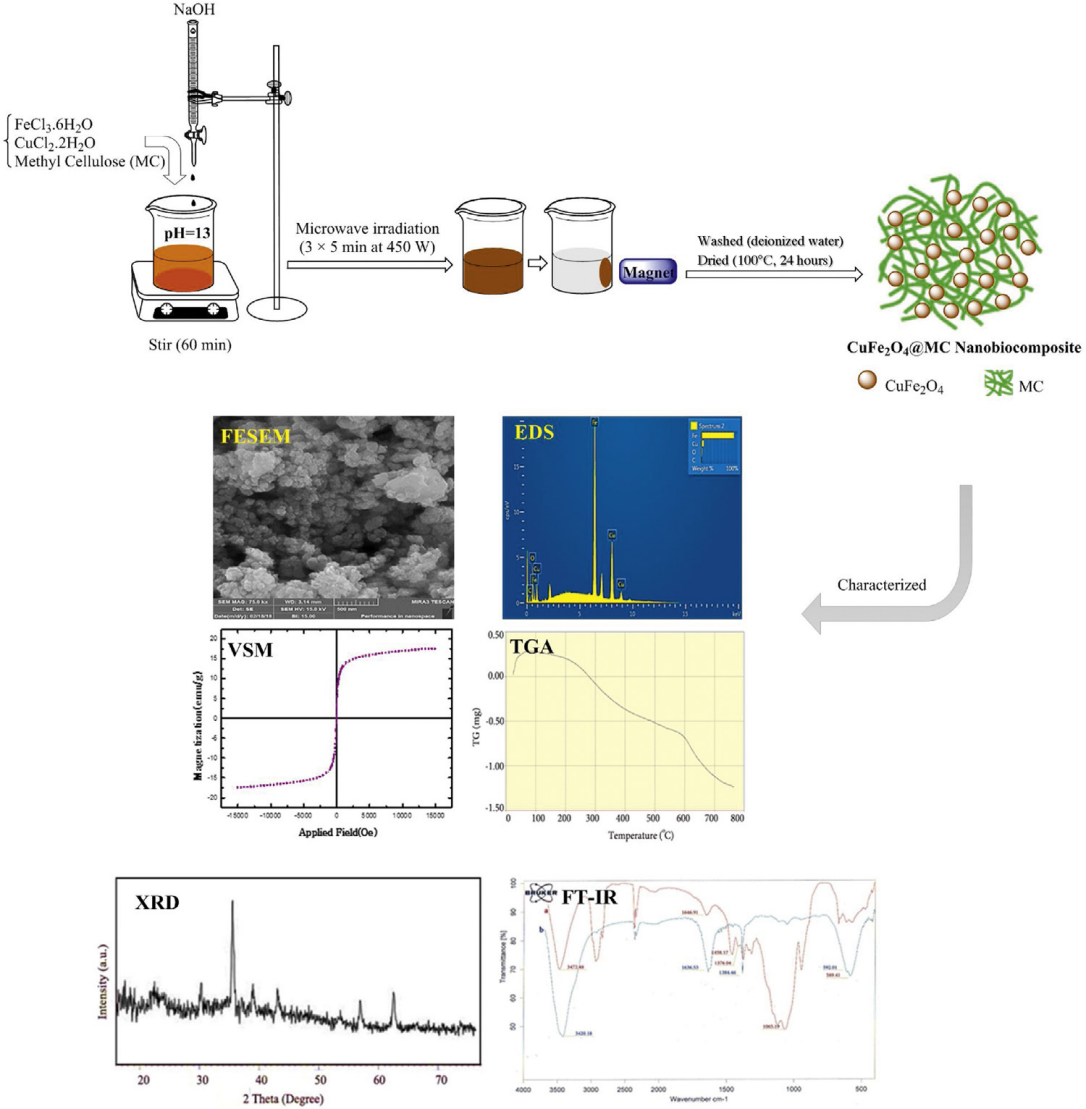
Synthesis and characterization of CuFe_2_O_4_@MC as a new magnetic nanobiocomposit.

In the first step, FeCl_3_.6H_2_O (20 mmol; 5.4 g), plus CuCl_2_.2H_2_O (10 mmol; 1.7 g), were dissolved in the 50 mL deionized water (DW). Then, MC (1 g) was added to the gained solution, and the mixture was stirred at the room temperature (25 °C). After that, sodium hydroxide was added to the suspension during 1 h. Dark brown solution was transferred to microwave oven (3 × 5 min at 450 W) (Samsung Microwave ME201, 20 L). Afterwards, CuFe_2_O_4_@MC as a lightweight sediment powder was observed. In the next step, the obtained nanobiomagnetics were separated by using an external magnet and was washed several times with DW. The final product was dried in an oven vacuum at 100 °C for 24 h [21]. The Fourier transform infrared spectroscopy (FT-IR) of the samples was measured using a FT-IR 6300 Brucker, and the X-ray powder diffraction (XRD) of CuFe_2_O_4_@MC was reported in the diffraction angle range of 2*θ* = 15^○^–70^○^ by an X'Pert PRO MPD PA nalytical using Ni-FILTERED Cu K*α* radiation. Thermal gravimetric analysis (TGA) was carried out using an STA (PC Luxx 409-NETZSCH) instrument at the rate of 10 °C min^−1^ in air. The CuFe_2_O_4_@MC magnetic properties were characterized with a vibrating sample magnetometer (VSM) (LakeShore Cryotronics-7404) at the room temperature (25 °C). The CuFe_2_O_4_@MC chemical composition, microstructure, and morphology were evaluated with field emission scanning electron microscope-energy dispersive spectroscopy (FESEM-EDS) (MIRA3TESCANXMU) (Fig. 2).

The dissolved Fe and Cu ions quantity in leachate was estimated with flame atomic absorption spectrometer (PG Instruments, model PG 990, England) at the wavelength of 248.30 nm and 324.70 nm, respectively. The CIP concentration was specified with HPLC device (Waters E600, USA) using column with the properties of C_8_; 259 × 4.6 × 5 mm with UV detector at wavelength of 272 nm with a hybrid mobile phase 10 micro molar HCl and acetonitrile (80:20 v/v), and input flow rate of 1 mL/min (Table 1). Chemical oxygen demand (COD) was determined by spectrophotometer (Shimadzu, Japan).

**Table 1:** Details of the HPLC analysis.

Characteristic	Condition
Detector	UV absorbance at the wavelength of 272 nm
Column characteristic	C_8_, 250 mm length and 4.6 mm internal diameter
Mobile phase	HCl:CH_3_CN (80:20, V/V)
Flow rate of mobile phase	1 mL/min
Volume of injection	20 μL

## Comparison of the photolysis, adsorption, and photocatalytic processes

In the researches which are about photocatalysis processe, comparison of the adsorption, photolysis results, and the photocatalytic mechanisms are valuable so, the mentioned processes were compered at irradiation time = 90 min, at pH = 7, CuFe_2_O_4_@MC = 0.2 g, and CIP concentration =3 mg/L [21]. The results indicated that the photocatalytic processes, adsorption, and photolysis are effective methods in the CIP removal process. The removal efficiency in the photolysis process with UV, the adsorption with CuFe_2_O_4_@MC, and the photocatalytic process was obtained 10.11 %, 17.6 % and 80.77 %, respectively. It means that the higher CIP removal efficiency is depends on the photocatalytic mechanism against photolysis and adsorption processes.

## Optimization of effective parameters on the CIP degradation

**Fig. 3:**
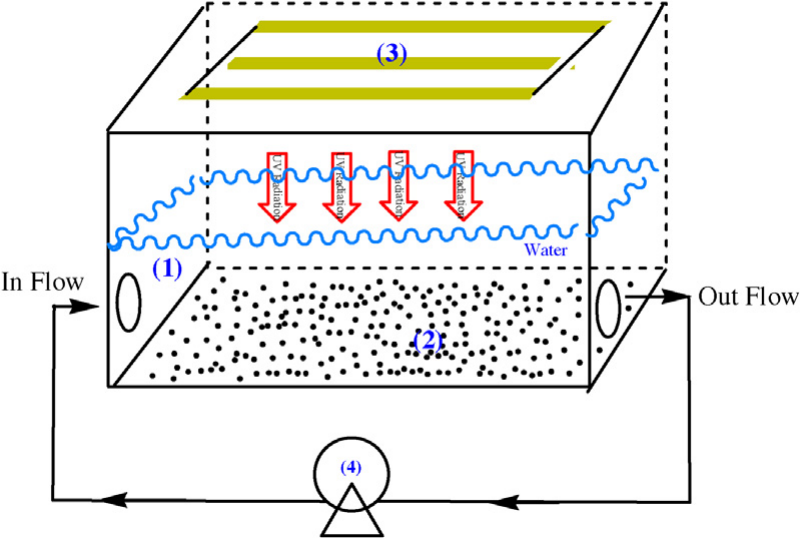
The designed photoreactor for the CIP photocatalytic degradation (1. The Plexiglas reactor, 2. photocatalyst, 3. UV-C lamp, and 4. Peristaltic pump).

Effects of initial CIP concentrations (3, 5, 7, and 9 mg/L), pHs (3, 7, 11), nanocatalyst loadings (0.025, 0.05, 0.1, 0.2, 0.3, and 0.4 g), and UV-C irradiation time (15, 30, 45, 60, 75, and 90 min) were optimized in a batch photoreactor. A plexiglas rectangular cubic shape photoreactor with internal dimensions of 25 cm (length), 10 cm (width), and 5 cm (height), an applicable volume of 300 mL was used in this study which had three UV-C lamps (low pressure, 6 W, Philips). The lamps were installed on the top of the reactor. To generate more hydroxyl radicals, a reactor was designed which could supply the minimum distance between the light supplier and the catalyst. To mix the reactor contents a peristaltic pump with a flow of 1 mL/s was used. The designed photoreactor is shown in Fig. 3. The samples were collected at the specific times during the irradiation. The samples were examined by HPLC when the CuFe_2_O_4_@MC was separated with an external magnet. After that, the degradation efficiency (*η*) was obtained by Eq. (1):

(1) *η*% = 100 (C_0_ - C_t_)/C_0_

Where C_t_ and C_0_ show the obtained CIP concentration at different periods of irradiation time (t) and at nil min, respectively, indicated by HPLC.

## Comparison between the CuFe_2_O_4_@MC and CuFe_2_O_4_ photocatalytic performance

The CuFe_2_O_4_@MC and CuFe_2_O_4_ photocatalytic performance in the CIP degradation was compared in the optimal conditions such as pH of 7, irradiation time of 90 min, CIP concentration of 3 mg/L, and photocatalyst loading of 0.2 g. The CIP degradation efficiency with these photocatalysts was obtained 80.77 % and 51.03 %, respectively.

## Kinetics of the CIP degradation

The CIP kinetics degradation were evaluated with the *pseudo-*first order (Eq. 2) and the *Langmuir-Hinshelwood* (Eq. 3) kinetic models (Table 2).

**Table 2:** The *pseudo-*first order and the *Langmuir-Hinshelwood* kinetic models.

Model	Formula	Parameters
*Pseudo-*first order	Ln (C_t_/C_0_) = -K_obs_t	C_0_ (mg/L): initial concentrations of CIP
		C_t_ (mg/L): CIP concentration at certain reaction times
		K_obs_ (min^−1^): constant rate of the *pseudo-*first order reaction
		t (min): irradiation time
*Langmuir-Hinshelwood*	$\frac{1}{K_{\text{o}\text{b}\text{s}\;}}=\frac{1}{K_{c}K_{L-H}}+\frac{C_{0}}{K_{c}}$	K_c_ (mg/L.min): constant rate of the superficial reaction
		K_L-H_ (L/mg): adsorption equilibrium constant of the *Langmuir-Hinshelwood* model

The kinetic linear models indicate that the CIP degradation, following the *pseudo*-first order kinetic model and the *Langmuir*-*Hinshelwood* equations. In accordance with the *pseudo*-first order kinetic model, the correlation coefficient (R^2^) for concentrations of 3, 5, 7, and 9 mg/L was 0.902, 0.923, 0.922, and 0.929, respectively. The *Langmuir-Hinshelwood* equilibrium adsorption coefficient and the superficial reaction rate constant were gained 0.202 L/mg and 0.141 mg/L min, respectively. The high correlation coefficient (R^2^ = 0.930) indicated that the CIP photocatalytic degradation followed the *Langmuir-Hinshelwood* kinetic model.

## Investigation of the reusability and chemical stability of CuFe_2_O_4_@MC

Considering the importance of the photocatalyst reusability in practical applications, the recycled CuFe_2_O_4_@MC photoactivity was examined. At first, The CuFe_2_O_4_@MC was separated with a magnet, and after that, was washed with ethanol/water and dried at 100 ^○^C for 2 h. The recycled photocatalyst in each run was added to a fresh solution of CIP the under UV-irradiation condition. In the first cycle, the CIP removal efficiency was gained 80.77 %. The results demonstrated that the CuFe_2_O_4_@MC photocatalytic activity had significant reduction in the second cycle (75.25 %) and maintained relative stability. Adsorption of intermediate products on the photocatalytic active sites can reduce the percentage of the CIP degradation. However, the CIP removal efficiency was obtained 73.78 % in the fourth cycle. Moreover, the Fe and Cu metal ions leaching in the photocatalytic process and loss of them could cause the CIP degradation efficiency reduction. Due to this reason, after the fourth cycle, copper and iron ion concentrations were determined in the solution. However, the mentioned ions were not detected in the solution. The CuFe_2_O_4_@MC chemical stability was determined with the XRD analysis, which showed the XRD peaks intensity after the fourth cycle, did not have an obvious change. This result cause to understand that this photocatalyst has good chemical stability and could be recycled.

## Conclusion

Briefly, a strong, magnetically separable photocatalyst was prepared by a simple, fast, green, and new microwave-assisted method and by using iron and copper salts on MC in an alkali medium. The magnetic nanobiocomposite characterization showed pure phase spinel ferrites, spherical particle morphology with smaller agglomeration, and the ferromagnetic nature of CuFe_2_O_4_@MC. The maximum CIP removal efficiency occured in the optimum conditions at pH 7, CIP concentration 3 mg/L, photocatalyst loading 0.2 g, and irradiation time 90 min. Then, after being used in the fourth cycle CuFe_2_O_4_@MC was separated with a magnet and recycled without loss considerable photocatalytic activity. CuFe_2_O_4_@MC has a lot of advantages such as high reusability, stability, environmentally-friendly and great photocatalyst activity. As well as, it can be applied for the contaminated water treatment.
